# Identification of New Rofecoxib-Based Cyclooxygenase-2 Inhibitors: A Bioinformatics Approach

**DOI:** 10.3390/ph13090209

**Published:** 2020-08-26

**Authors:** Rozires P. Leão, Josiane V. Cruz, Glauber V. da Costa, Jorddy N. Cruz, Elenilze F. B. Ferreira, Raí C. Silva, Lúcio R. de Lima, Rosivaldo S. Borges, Gabriela B. dos Santos, Cleydson B. R. Santos

**Affiliations:** 1 Graduate Program in Medicinal Chemistry and Molecular Modeling, Health Science Institute, Federal University of Pará, Belém 66075-110, PA, Brazil; lrozires@gmail.com (R.P.L.); raics@usp.br (R.C.S.); luciorolima@gmail.com (L.R.d.L.); lqfmed@gmail.com (R.S.B.); 2 Laboratory of Modeling and Computational Chemistry, Department of Biological and Health Sciences, Federal University of Amapá, Macapá 68902-280, AP, Brazil.; josianeviana2007@gmail.com (J.V.C.); vilhenac@hotmail.com (G.V.d.C.); jorddynevescruz@gmail.com (J.N.C.); elenilze@yahoo.com.br (E.F.B.F.); 3 Laboratory of Organic Chemistry and Biochemistry, University of State of Amapá, Macapá 68900-070, AP, Brazil; 4 Department of Chemistry, Faculty of Philosophy, Sciences and Letters of Ribeirão Preto, University of São Paulo, Ribeirão Preto 14090-901, SP, Brazil; 5 Institute of Collective Health, Federal University of Western Pará, Santarém 68040-255, PA, Brazil; gabiasa@hotmail.com

**Keywords:** anti-inflammatory, cyclooxygenase-2, rofecoxib, bioinformatics

## Abstract

The cyclooxygenase-2 receptor is a therapeutic target for planning potential drugs with anti-inflammatory activity. The selective cyclooxygenase-2 (COX-2) inhibitor rofecoxib was selected as a pivot molecule to perform virtual ligand-based screening from six commercial databases. We performed the search for similarly shaped Rapid Overlay of Chemical Structures (ROCS) and electrostatic (EON) compounds. After, we used pharmacokinetic and toxicological parameters to determine the best potential compounds, obtained through the softwares QikProp and Derek, respectively. Then, the compounds proceeded to the molecular anchorage study, which showed promising results of binding affinity with the *h*COX-2 receptor: LMQC72 (∆G = −11.0 kcal/mol), LMQC36 (∆G = −10.6 kcal/mol), and LMQC50 (∆G = −10.2 kcal/mol). LMQC72 and LMQC36 showed higher binding affinity compared to rofecoxib (∆G = −10.4 kcal/mol). Finally, molecular dynamics (MD) simulations were used to evaluate the interaction of the compounds with the target *h*COX-2 during 150 ns. In all MD simulation trajectories, the ligands remained interacting with the protein until the end of the simulation. The compounds were also complexing with *h*COX-2 favorably. The compounds obtained the following affinity energy values: rofecoxib: ΔGbind = −45.31 kcal/mol; LMQC72: ΔGbind = −38.58 kcal/mol; LMQC36: ΔGbind = −36.10 kcal/mol; and LMQC50: ΔGbind = −39.40 kcal/mol. The selected LMQC72, LMQC50, and LMQC36 structures showed satisfactory pharmacokinetic results related to absorption and distribution. The toxicological predictions of these compounds did not display alerts for possible toxic groups and lower risk of cardiotoxicity compared to rofecoxib. Therefore, future in vitro and in vivo studies are needed to confirm the anti-inflammatory potential of the compounds selected here with bioinformatics approaches based on rofecoxib ligand.

## 1. Introduction

**Figure 1:**
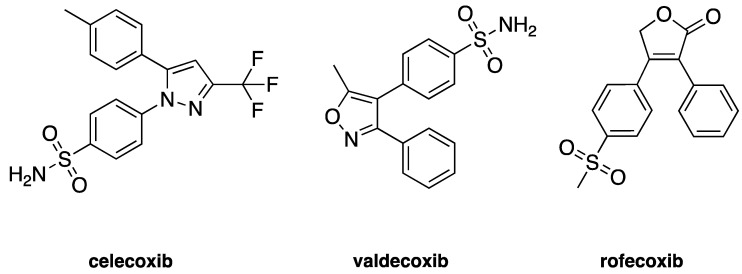
Two-dimensional (2D) chemical structures of cyclooxygenase-2 (COX-2) inhibitors celecoxib, valdecoxib, and rofecoxib.

Cyclooxygenases are an important and thoroughly studied group of enzymes present in two isoforms in mammals: constitutive form cyclooxygenase-1 (COX-1) and an inducible form cyclooxygenase-2 (COX-2) [1]. The COX-1 enzyme is expressed in most tissues and is responsible for maintaining homeostasis and production of prostaglandins (PGs) [2]. COX-2 is found predominantly in the brain, renal, and endothelial cells and is significantly increased through various acute and chronic inflammatory infections [3,4]. The inhibition of the COX-2 enzyme through selective anti-inflammatory drugs has been an important strategy to contain the inflammation process. Many selective COX-2 inhibitors achieve the market as anti-inflammatory drugs, such as celecoxib (Celebrex^®^), valdecoxib (Bextra^®^), and rofecoxib (Vioxx^®^) [5], once it was thought that more selective drugs leads to less gastric side-affects—see Figure 1. Nevertheless, some of these selective inhibitors of COX-2 also depress prostacyclin (PGI2), an atheroprotective agent, which might predispose patients to heart attack and stroke [6]. 

Thus, the side effects promoted by these therapeutic agents directed the search for new compounds, whose anti-inflammatory potential is accompanied by greater selectivity and specificity, minimal side effects, and lower cost [7].

Rofecoxib (Vioxx^®^) was approved by the Food and Drug Administration (FDA) for human use in May 1999, and withdrawn from the market on September, 2004 [8]. This drug, from the Coxibs family, presents risks of cardiovascular events; however, it presents anti-inflammatory effects and properties similar to traditional non-steroidal anti-inflammatory drugs (NSAIDs) with reduced gastrointestinal toxicity, which would have the potential [5,6,7,8,9] without side-effects, such as ulcers and gastrointestinal problems [10]. Since then, rofecoxib became an important prototype for the design of new promising NSAIDs for the COX-2 target and with possible minor side effects in humans [11].

**Figure 2:**
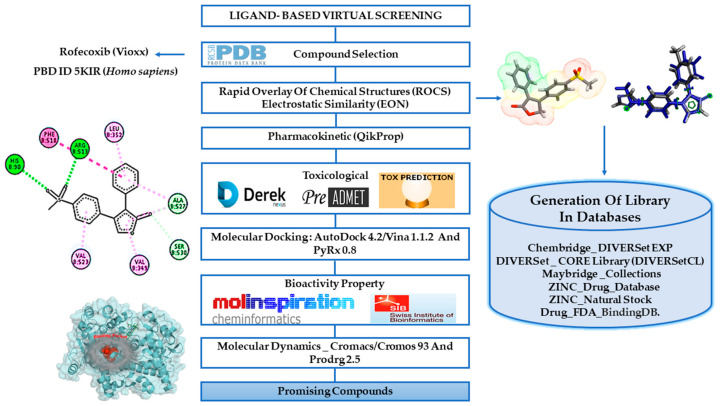
Workflow summarizing the methodological steps.

In this work, we used a virtual screening ligand-based methodology to identify new potential COX-2 inhibitors based on the rofecoxib structure [12,13,14]. The virtual screening strategy was chosen once it has been widely applied in the early phase of drug discovery, being able to accelerate hit discovery and reducing drug development costs. Thus, the similarity and electrostatic potential of the selected structures were performed using computer programs and commercial databases of compounds [15] and then we performed the filtering of the results considering the pharmacokinetic and toxicological properties [16,17,18,19,20]. Furthermore, the docking simulation evaluated the binding affinity of compounds to COX-2 in comparison with rofecoxib [20,21]. Biological target prediction was used as a screening step through the web server Swiss [22] and the bioactivity was determined on the Molinspiration web server [23]. Finally, we used molecular dynamics to investigate interaction over time in COX-2 of the promising compounds. General scheme of the methodological steps in this article is presented in Figure 2 (see more details in the Materials and Methods section).

## 2. Results and Discussion

### 2.1. Ligand-Based Virtual Screening

In this initial stage, the pivot molecule rofecoxib was used as a research model for the virtual screening in six commercial molecule databases: Chembridge DIVERSetEXP, DIVERSet CORE Library (https://www.chembridge.com) [24], Maybridge Collections (www.maybridge.com) [25,26], ZINC Drug Database, ZINC Natural Stock (http://zinc.docking.org) [27], and Drug FDA BindingDB (http://www.bindingdb.org) [27] using the programs Rapid Overlay of Chemical Structures (ROCS) and electrostatic similarity (EON).

In the ROCS software [28,29,30], we used a virtual screening tool for searching three-dimensional (3D) structures with chemical similarity and shape with the pivot molecule rofecoxib [16,28]. The rofecoxib molecule was used as a comparison model with each of the molecules in the databases looking for chemical similarity [16,31], according to the structural characteristics and molecular volume fractions of the pivot molecule, observing the maximum overlap in relation to the shape (chemical structure), using as a parameter the Gaussian functions [32] implemented in the ROCS software. The compounds were selected and classified by means of an algorithm that generated relative scores for the overlapping of forms in the databases according to the pharmacophoric characteristics of rofecoxib [15,33]. This stage of virtual screening identified the most similar two thousand (2000) molecules in each database (Top_2000), resulting in twelve thousand (12,000) tracked structures, which exhibited highest scores of chemical similarities.

In the sequence, the selected compounds were submitted to electrostatic correlations of aligned molecules based on the Tanimoto electrostatic score in EON software [34,35]. This electrostatic potential is calculated using OpenEye’s Poisson-Boltzmann (PB) electrostatic calculation [33,36]. The Top 100 molecules by database (Top_100), led to six hundred structures (600) hits with best alignment based on the electrostatic potential [15]. 

The remaining six hundred structures (600) were then evaluated for their pharmacokinetic properties (the absorption, distribution, metabolism) using the QikProp software [37,38,39]. Structures submitted for pharmacokinetic study, resulted in two hundred and thirty-three (233) hits that presented satisfactory pharmacokinetic properties, especially electronic affinity, Lipinski’s rule, and the central nervous system (CNS) parameter, when compared with the properties of Rofecoxib.

The “surviving structures” were submitted to DEREK software [40] to evaluate toxicological properties, having as reference the properties of the commercial drug Rofecoxib. Thus, only seventy-nine structures were selected because they did not present toxicity alerts and toxicophoric groups [21]. Subsequently, these structures were subjected to a molecular study to assess binding mode and affinity with *h*COX-2 receptor. At the end of this process, only three structures (LMQC72, LMQC36, and LMQC50) were selected, for having binding affinity with the COX-2 molecular target and good pharmacokinetic and toxicological profile. Therefore, this study discusses the main selected structures (LMQC72, LMQC36, and LMQC50) that offer promising results with the therapeutic ligand of interest.

### 2.2. Pharmacokinetic Predictions for the Selected Compounds 

In silico prediction of absorption, distribution, metabolism, excretion, and toxicity (ADMET) properties are fundamental for the selection of the most promising molecules for further development. The selected structures were subjected to predictions of pharmacokinetic properties absorption, distribution, metabolism, and elimination using the QikProp software. To evaluate these properties, nine parameters (see Table 1) were used, related to the inflammatory process, and based on the compound Rofecoxib.

The #star parameter compares results obtained with properties of drugs present in database of the QikProp software [37]. An alert is given when a result is outside the 95% range of values similar to commercially available drugs. This parameter takes into account a set of properties and descriptors such as: molecular weight (MW), dipole moment, electron affinity (EA), total solvent accessible surface area (SASA), hydrophobic component of the SASA (FOSA), hydrophilic component of the SASA (FISA), π (carbon and attached hydrogen) component of the SASA (PISA), weakly polar component of the SASA (halogens, P, and S) (WPSA), polar surface area (PSA), molecular volume, number of rotatable bonds (#rotor), number of hydrogen bond donor groups (donorHB), number of hydrogen bond acceptor groups (accptHB), predicted polarizability in cubic angstroms (QPpolrz), predicted hexadecane/gas partition coefficient (QPlogPC16), predicted octanol/gas partition coefficient (QPlogPoct), predicted water/gas partition coefficient (QPlogPw), predicted octanol/water partition coefficient (QPlogP*o*/*w*), predicted aqueous solubility (logS), prediction of binding to human serum albumin (QPLogKhsa), predicted brain/blood partition coefficient (QPlogBB), number of likely metabolic reactions (#metabol) [38]. These results for the three selected compounds are shown in Table 1.

The pharmacokinetic predictions for LMQC72, LMQC35, and LMQC50 show no violations in the descriptors and properties analyzed, which indicates that its properties are similar to commercial drugs (#star = 0). However, rofecoxib has an alert (#star = 1) in the molecular descriptor electronic affinity (EA), which is out of range (−0.9 to 1.7), with a value of 1.99 eV. EA is an essential characteristic for intermolecular interactions and charge transfer complex [41,42,43,44]. 

Lipinski’s (RO5) investigation are based on molecular weight (MW), lipophilicity (represented by the partition coefficient, LogP) and hydrophilicity (represented by the number of hydrogen bond donors and acceptors groups) descriptors. RO5 represents a well-established form of limits for the absorption and permeability of a drug [45]. In this study, LMQC72, LMQC36, and LMQC50 showed no violations to RO5, indicating that these compounds would make it a likely orally active drug in humans. Rofecoxib is an orally administered drug and in consonance, its properties did not violate the rule of Lipinski (RO5). Thus, this result predicts similarity to biological activity designed for oral administration [37,46].

The percentage of human oral absorption (%HOA) was evaluated through a set of properties based on number of metabolites (#metab), number of rotating bonds (#rotor), solubility and cell permeability in comparison within the standards [38]. The prediction %HOA of the selected compounds showed excellent results, once LMQC72, LMQC36, and LMQC50 exhibited values of 100% HOA. Moreover, rofecoxib showed a value of 82.40% HOA, which indicates a better oral absorption of the novel compounds.

The apparent permeability between octanol/water (QplogP*o*/*w*) is a parameter used in drug design processes to estimate solubility, membrane permeability, and bioavailability [47,48,49,50]. The calculated values regarding QlogP*o*/*w* for LMQC72, LMQC36, and LMQC50 are higher than the value found for rofecoxib (QplogP*o*/*w* = 1.45). LMQC72, LMQC36, and LMQC50 values ranged from 2.18 ≥ QlogP*o*/*w* ≥ 4.21, considered more lipophilic compounds (logP*o*/*w* ≥ 0). Thus, this means that the novel compounds are mainly absorbed by passive transcellular processes in the intestine. LMQC72, LMQC36, and LMQC50 are within the limits indicated in ranges 2 to 5, favoring better absorption, that is, easily overcome the lipid bilayer of biological membranes [51].

Models predictive of intestinal drug absorption are important in drug development to identify compounds with promising biopharmaceutical properties [52]. In this study, the intestinal absorption was estimated by Caco-2 and Madin-Darby canine kidney (MDCK) cell values [53]. Predictions values of these cells make it possible to evaluate the cell permeability of potential drug candidates and routes of drug transport (e.g., passive versus carrier mediated) [54,55]. Descriptors used for the prediction of passive transport should have values above 500 nm/s to be considered good, whereas values less than 25 nm/s are considered poor. LMQC72, LMQC36, and LMQC50 showed values between 1470.77 and 1751.71 nm/s for Caco-2 cells and between 900.66 and 3415.52 nm/s for MDCK cells. Thus, the compounds showed good results, indicating a promising intestinal absorption and even better in comparison with rofecoxib.

The blood-brain barrier (BBB) is a critical factor in drug design. High penetration is needed for CNS-active drugs, while negligible penetration may be desirable in order to minimize CNS-related side-effects of drugs with a peripheral site of action [56,57] It is a selective barrier formed by narrow junctions between endothelial cells, to limit the penetration of different blood substances in the brain [56,58]. In our study, compounds LMQC71, LMQC36, and LMQC50 were evaluated by the brain-blood partition coefficient (QPlogBB). The parameter established to indicate inactivity for penetration into the blood-brain barrier and consequent CNS activity includes values below 1 (C_Brain_/C_Blood_ < 1) and, for values greater than 1, it suggests activity in the central nervous system [20,37]. Evaluation of the penetration capacity (QPlogBB) of the LMQC72, LMQC36, and LMQC50 exhibited negative values (<1) which reveals low penetrability to CNS [57]. 

Then, the prediction of the central nervous system activity of the selected compounds was performed. The established CNS activity parameter ranges from −2 (inactive) to +2 (active). In our study, LMQC72, LMQC36, and LMQC50 exhibited values equal to zero (0), which indicates that they are inactive and do not produce CNS side effects in humans [38]. Therefore, these results are similar to the pivot compound rofecoxib, which has values of below 1 (inactive) for the parameters: QPlogBB and CNS. 

In terms of pharmacokinetic properties, one may evaluate that the new *h*COX-2 inhibitors show better pharmacokinetic performance without violations in their descriptors and molecular properties when compared to rofecoxib.

### 2.3. Molecular Docking Simulations Study

**Figure 3:**
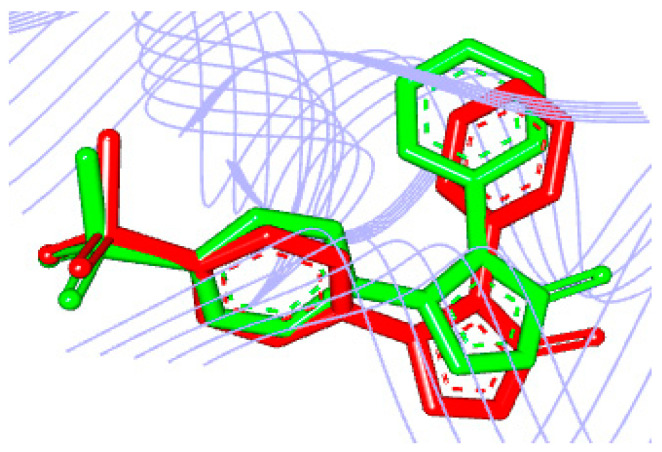
Overlapping of the crystallographic ligand (**green**) and the conformation obtained by re-docking (**red**).

The seventy-nine compounds selected here by the toxicological studies followed the study of molecular docking to assess the binding mode and affinity with the *h*COX-2 receptor. To validate the molecular docking protocol, the crystallographic ligand was re-docked in the *h*COX-2 with the Protein Data Bank (PDB) ID 5KIR structure with resolution 2.69 Å [14]. The root mean square deviation (RMSD) obtained by re-docking, and the bonding pose found in the complex was 0.98 Å [15]. The comparison between the crystallographic ligand and the pose predicted by docking overlap of the ligand can be visualized in Figure 3. According to literature, the binding mode prediction using docking should present RMSD value <2.0 Å when superimposed to the crystallographic pose of the ligand [20,59,60].

We also evaluated the interaction affinity of rofecoxib to *h*COX-2. The binding affinity value obtained in re-docking was ∆G = −10.4 kcal/mol. It was considered close to the experimental value (∆G = −9.2 kcal/mol). Thus, our protocol showed satisfactory performance in predicting the interaction conformation once the interaction affinity value was close to the observed experimentally, see Table 2.

Protein-ligand binding affinity is essential for biological processes, as these physical and chemical interactions determine biological recognition at the molecular level. In this way, it is possible to look for a ligand capable of inhibiting or activating a specific target protein through its interaction. In such a way, it is important to find a ligand that binds to a target protein with high affinity [61]. 

**Figure 4:**
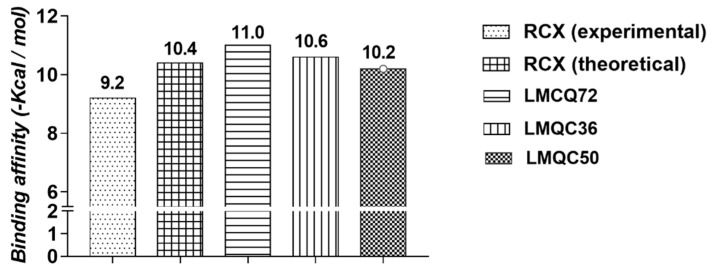
Binding affinity values (Kcal/mol) of the most promising compounds to *h*COX-2.

All 79 compounds that showed good pharmacokinetic and toxicological profiles were subjected to the molecular docking simulations in order to verify the binding affinity at the target receptor binding site (*h*COX-2, PDB 5KIR). Binding affinity values of the compounds with higher affinity to *h*COX-2 compounds are shown in Figure 4.

Molecular docking of LMQC72 (∆G = −11.0 kcal/mol) and LMQC36 (∆G = −10.6 kcal/mol), have the most negative binding affinity values when compared to rofecoxib (∆G = −10.4 kcal/mol), indicating a stronger binding based on the values of binding affinity. LMQC50 present a binding affinity value (∆G = −10.2 kcal/mol) close to the pivot rofecoxib; thus, showing a satisfactory binding affinity value, see Table 3 and Table 4.

Molecular docking studies (AutoDock/Vina) [62] also allowed us to determine the types of interactions between the target receptor’s binding site with the promising compounds LMQC72, LMQC36 and LMQC50. Table 3 shows the interactions between the *h*COX-2 inhibitor rofecoxib (PDB ID 5KIR). In comparison with the pivot, Table 4 shows the types of interactions and amino acid residues between *h*COX-2 and LMQC72, LMQC36, and LMQC50. 

**Figure 5:**
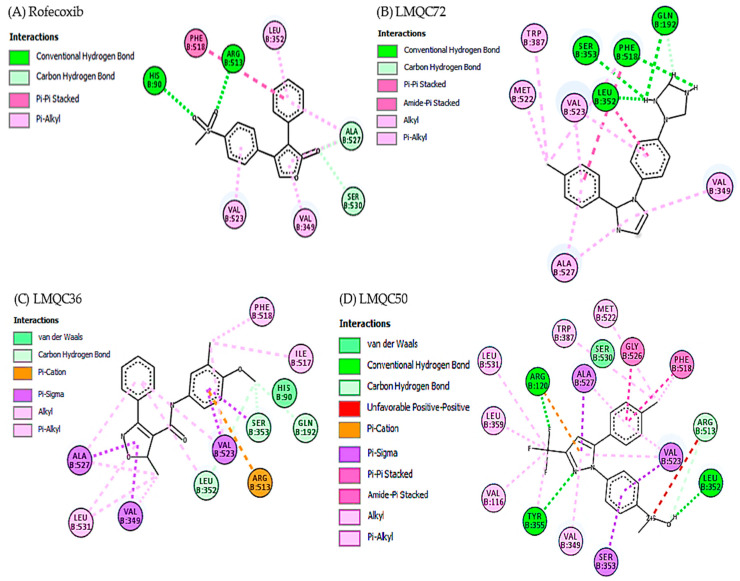
Interactions of the active site of the *h*COX-2 with rofecoxib (**A**); LMQC72 (**B**); LMQC36 (**C**); and LMQC50 (**D**). Figure generated using AutoDock/Vina software.

Crystallographic complex of rofecoxib with *h*COX-2 deposited in the PDB under the code 5KIR exhibits the main interactions in the region of monomer B of the protein. Rofecoxib methyl sulfone group binds to the active site of the enzyme, specifically with residues: His90 and Arg513 in the α helix of the hydrophilic part of *h*COX-2 (chain B) [14,63]. Figure 5A shows the amino acid residues Phe518, Leu352, Ala527, Ser530, Val349, and Val523 of *h*COX-2 interacting with rofecoxib [14].

Experimental data shows that the selected compounds share the following interactions with *h*COX-2: LMQC72 makes a hydrophobic and Pi-Alkyl type interaction with residues Val523 and Ala527, respectively in the α-helix and β-leaf regions of the protein (Figure 5B). LMQC36 makes a hydrophobic and Pi-Alkyl interactions with Ala527 residue located in the α-helix region of the protein (Figure 5C). LMQC50 interacts with three amino acid residues that are present in the *h*COX-2 interaction with rofecoxib, which are Val349 (Pi-Alkyl), Phe518 (Pi-Pi Stacked) and Arg513 (hydrogen bond), in the α-helix region of the protein (Figure 5D). 

Therefore, the evaluation carried out through the AutoDock Vina program enables us to affirm that the selected compounds are close to the interactions made with the rofecoxib (RCX) ligand (5KIR) at the active site of *h*COX-2, as the molecules share the main interactions in the hydrophobic part with the and amino acid residues Val523, Val349, Ala527, Phe518, and Arg513 linked by B chain. 

### 2.4. Biological Target Prediction

Table 5 summarizes the chemical information from the selected structures resulting from the molecular docking study. The three remaining compounds were subjected to the bioactivity prediction, through the Molinspiration server (https://www.molinspiration.com/). In this prediction, biological activity measured by the bioactivity score for enzyme inhibitor was evaluated enzyme (see Table 6), which are classified into three different ranges: molecule having bioactivity score more than 0.00 is most likely to possess considerable biological activities, while values −0.50 to 0.00 are expected to be moderately active, and if score is less than −0.50, it is presumed to be inactive [21].

The bioactivity scores of the LMQC72, LMQC36, and LMQC50 structures were calculated for different parameters, as receptor binding of the ligand to the G protein coupled (GPCR) and nuclear receptor ligand, modulating ion channel, kinase inhibition, protease inhibition, and inhibition of enzyme activity. Then, compared with the bioactivity score of the pivot molecule rofecoxib [23].

The bioactivity scores for the G protein-coupled receptor ligand (GPCR) are most active for the LMQC72, LMQC50 and rofecoxib structures with values greater than 0.00. Meanwhile, the LMQC36 has a moderately active score between −0.5 to 0.00. The score values of the LMQC 72, LMQC36, and LMQC50 structures are considered good because they are close to the pivot compound with probable biological activity (see Table 6). This estimated property, the binding to the ligand by the GPCR receptors, act as the main responsible for the mediation of inflammatory (and anti-inflammatory) responses and can contribute to the regulation of the vascular permeability process [65].

The results of the ion channel modulators’ scores for the LMQC36, LMQC50, and rofecoxib structures are estimated score values between −0.50 to 0.00 considered moderately active and the LMQC72 structure with a score value above 0.00 considered biologically active. These ionic modulators are important for planning potential anti-inflammatory drugs because they participate in the protection of tissues against lesions induced by the inflammatory process, they carry charged particles across cell membranes and their activity can be directed towards the discovery of new potential drugs for the regulation of the depolarization of ionic charges [66,67].

The LMQC72 and LMQC50 structures have score values for kinase inhibitors greater than 0.00 considered biologically active. Meanwhile, the compound rofecoxib and LMQC36 have moderately active score values (see Table 5) for protein kinase inhibitors, as cyclooxygenase-2 is induced by various extracellular signals including pro-inflammatory stimuli and growth promoters. A cyclooxygenase-2 is induced by several extracellular signals, including pro-inflammatory and stimulating growth promoters. Thus, all of the signals converge for the activation of mitogen-activated protein kinases (MAPK) that regulate cyclooxygenase-2 mRNA and contribute to the infection treatment process [68].

Moreover, the nuclear receptor score values (NRs), in the LMQC72 and LMQC36 structures, are considered moderately active, as they have score values between −0.5 to 0.00. LMQC50 and rofecoxib are considered biologically active, with a score value above 0.00, according to the classification ranges of Smant and Chowdhary. The bioactivity of nuclear receptors (NRs) is important because they are involved in several physiological processes, including homeostasis, an important process that regulates inflammation [69].

The LMQC72, LMQC36, LMQC50 structures have moderately active score values between −0.5 and 0.00 for protease inhibitors. Already, the dynamic compound of rofecoxib has an estimated value greater than 0.00 considered active. Therefore, the results of the LMQC72 and LMQC50 structures are considered to have biological activity (active), enzyme inhibitor, since they had score values greater than zero, such as the compound Rofecoxib. While LMQC36 is expected to be moderately active with a score between −0.50 to 0.00. The activity score profile of the selected structures demonstrates the probability that they are biologically active and that they have the necessary properties to act with potential enzyme inhibitors of cyclooxygenase-2 (COX-2) [70].

Compounds LMQC72, LMQC36, and LMQC50 were also submitted to web server Swiss Target Prediction (http://www.swisstargetprediction.ch) [22]. To identify the likelihood of bioactivity through similarity based on chemical structure and molecular form (Electroshape) [71]. The server uses a database of molecules: ChEMBL [72,73], DrugBank [74], PubChem [75], and ZINC [76] to track sets of molecules and identify proteins with ligands similar to bioactive molecules and also uses species selection for virtual screening (Top_25 *Homo sapiens*). The results of the virtual bioactivity screening for the enzymatic target performed by the Swiss TargetPrediction [77] server issued a summary displayed in percentages with the probability of being the enzymatic target [78].

The Table 5 shows the percentage probability values for enzyme inhibition. Prediction analysis of enzymatic inhibition for rofecoxib was 32%; while the selected compounds exhibited the following probability of reaching the enzyme: LMQC72 16%, LMQC36 8%, and LMQC50 4%. Thus, it is observed that rofecoxib did not reach 100% of the enzyme and the selected compounds had lower enzyme values. However, the results were assessed as likely for possible bioactivity and the structures proceeded with analysis taking into account the pharmacokinetic and toxicological profiles in which they presented favorable results.

### 2.5. Molecular Dynamics (MD) Simulations and Structural Analysis of Systems 

To evaluate the conformational changes in the receptor-ligand complexes along the time, the MD simulations were applied in 150 ns simulation nodes, for each complex *h*COX and ligand: rofecoxib, LMQC72, LMQC36, and LMQC50. The simulations also allowed the evaluation of the conformational changes in the structure of the ligand and the protein backbone. These conformational changes in the backbone and ligand were evaluated from the root mean square deviation plot (RMSD). 

**Figure 6:**
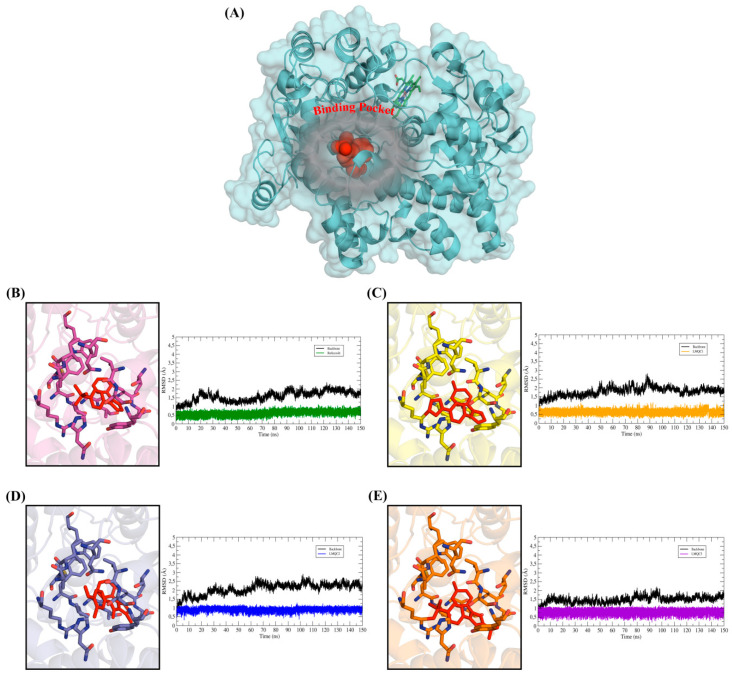
Shows the root mean square deviation (RMSD) plot along the path of molecular dynamics (MD) simulations. RMSD graphs for 150 ns of MD simulations. In all the Figures, the RMSD plot of the *h*COX-2 backbone was represented by the color black, while the RMSD of the ligands was represented in different colors. (**A**) General view of the protein structure with emphasis on the binding pocket occupied by the ligands. The protein was represented in cyan color and ligand protein was represented in spheres (red color). (**B**) RMSDs of *h*COX-2-rofecoxib- system, (**C**) RMSDs of *h*COX-2-LMQC72 system, (**D**) RMSDs of *h*COX-2- LMQC36 system, (**E**) RMSDs of *h*COX-2- LMQC50 system. Ligands and residues were represented in sticks.

To plot the RMSD of the backbone, Cα atoms were used, while to plot the RMSD of the ligand, all heavy atoms were used. In addition, the fluctuation of the residues from the protein backbone was evaluated, for this, the Cα atoms were also used. This analysis was performed to evaluate the difference in the structural fluctuation of the protein during the interaction with the different ligands, throughout the 150 ns MD simulation (see Figure 6) [25,79,80,81,82,83].

The RMSD plot reveals that the ligands showed small conformational variations when interacting with the protein along the time. Their RMSD graphs show slight variations, which suggests that the ligands remained interacting with the active site of the protein undergoing minor conformational changes. This conformational stability over 150 ns of MD simulations demonstrates a good interaction of the ligands with the molecular target, thus, remained in a favorable conformation to inhibit the biological receptor.

The low RMSD fluctuation of the ligands is also related to the interactions established in the binding pocket. All ligands showed interactions with residues observed in the results of molecular docking, which are summarized in Table 2 and Table 3. These interactions were able to keep the ligands interacting with the active site throughout the entire trajectory, allowing the maintenance of the receptor-ligand.

**Figure 7:**
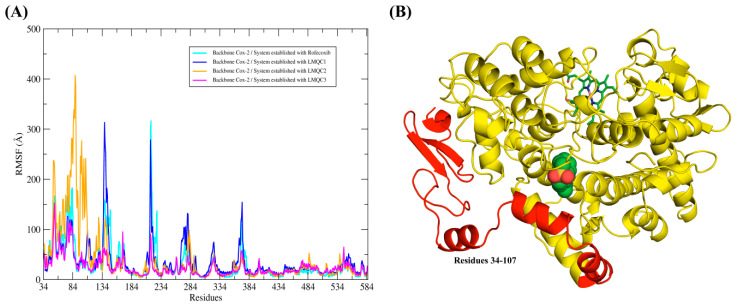
Shows the profile of the root-mean-square fluctuation (RMSF) plot for the *h*COX-2 protein backbone that was extracted from the molecular dynamics trajectories. RMSF plot overlay for all complexes. (**A**) Profile of the RMSF graphs and (**B**) Representation of the protein, where the residues (37–107) that showed the greatest fluctuations were highlighted in red color.

The different ligands were able to impact the flotation of the atoms of the *h*COX-2 backbone in different ways, as can be seen from the differences of the root-mean-square fluctuation (RMSF) plot. The greatest differences in fluctuations in protein residues are observed at residues 34–107. This region of the protein corresponds to the N-terminal portion; in addition, it is initially composed of a small alpha-helice, followed by two beta-leaves that will connect to another alpha-helice through a region’s relatively large loop. Finally, the residue gap is formed by three more alpha helices that are connected by loop regions (see Figure 7).

The greatest fluctuation of residues 34–107 was observed in the complex established with the LMQC36 ligand. Apparently, this greater fluctuation should impair the stability of the ligand at the active site, since residues 34–107 correspond to a region of the protein that is close to the active site. However, this behavior was not observed, since the RMSD plot of the ligand shows that the maintenance of the molecule in the binding pocket with conformational stability along the 150 ns trajectory. Additionally, the affinity energy value (ΔGbind = −36.10 kcal/mol) demonstrates that the ligand was able to interact favorably with the protein. This result demonstrates that this region of the protein, despite showing high fluctuation, was not able to impair the interaction of the ligand with the active site. This suggests that the residues around the active site are sufficient to keep the ligands complexed to the protein, despite fluctuations conformations observed in the region of the protein formed by the residues 34–107.

#### Binding Free Energy

To evaluate the interaction energy of the selected compounds with *h*COX-2, the Molecular Mechanics/*G*eneralized Born Surface Area (MM/GBSA) method was applied and the obtained results are summarized in Table 7. 

According to the values of affinity energy (ΔGbind), all ligands selected by molecular docking are able to establish stable complexes with *h*COX-2. Rofecoxib achieved the free energy value of ΔGbind= −45.31 kcal/mol. The other compounds reached the following affinity energy values: LMQC72: ΔGbind = −38.58 kcal/mol; LMQC36: ΔGbind = −36.10 kcal/mol; and LMQC50: ΔGbind = −39.40 kcal/mol. The compounds LMQC72, LMQC36, and LMQC50 showed favorable values of affinity energy for formation of the complexes. Van der Waals (ΔEvdW) interactions showed the greatest contributions to the formation of the different systems of this study. In addition, electrostatic (ΔEele) and non-polar (ΔGNP) interactions also contributed to complexes being formed spontaneously. The values of affinity energy for the three selected compounds were promising, as the values were relatively close to the obtained for rofecoxib. This demonstrates that the selected substances can be considered as putative *h*COX-2 inhibitors, being promising leads for new anti-inflammatory drugs project.

### 2.6. Structure-Activity Relationship of the Promising Molecules

The chemical structures of COX-2 inhibitors are heterogenic and can be classified into tricyclics and non-tricyclics compounds. Contrary to the classic NSAIDs, this new class of enzyme inhibitors is lacking a carboxylic group, thus effecting COX-2 affinity by a different orientation within the enzyme without formation of a salt bridge in the hydrophobic channel of the enzyme [6].

Celecoxib, Rofecoxib, Valdecoxib share in common the same structural features of the selected compounds LMQC36, LMQC50, and LMQC72, which exhibit a tricyclic scaffold, and a 1,2-diarylsubstitution on a central hetero ring system. In addition, these compounds show characteristic groups on one of the aryl rings that plays a crucial role on COX-2 selectivity. All selected compounds present five membered core heterocycles, even though all different from rofecoxib, which shows a furanone ring (see Figure 1).

**Figure 8:**
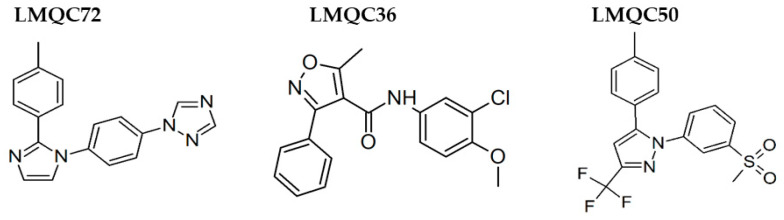
Promising bioactive compounds.

Compound LMQC72 present a pharmacophore-based 1,2,4 triazole group, which increases a certain degree of conformational rigidity to compound, which can be seen in the binding free energy essay. LMQC50 shows a pyrazole moiety, the same core as celecoxib (Figure 1 and Figure 8), which favors a hydrogen bond interaction with *h*COX-2 (Figure 5B). Moreover, LMQC50 presents a 4-sulfonylmethylphenyl substitution at 1 position on the pyrazole ring which increases the inhibitory effects against COX-2 enzyme [84]. 

LMQC36 shows an isoxazole ring such as valdecoxib (Figure 1 and Figure 8), linked to the aryl ring by an amide group, which also confers rigidity to the structure and favors an additional Pi-sigma interaction with hCOX (Figure 5C). Compound LMQC72 present an imidazole ring, favoring a Pi-Pi stacking interaction with *h*COX-2 (Figure 5D) [85].

According to previous studies, imidazole, triazole, ozaxol, benzene sulfonamide, and pyrazole favors the formation of hydrogen bonds capable of introducing a certain degree of conformational rigidity, indicating a wide range of pharmacological activity as a desired, anti-inflammatory activity (Figure 8) [86,87].

Due to the aforementioned facts, the compounds with the most promising results (Figure 8), were submitted to an investigation in SciFinder^®^, available on the internet, and linked to the Chemical Abstract Service (CAS) (https://scifinder.cas.org/), in order to verify additional information about structures and/or experiments with biological activities (patents). No additional information on the promising structures was found in the search. This demonstrates that the molecules mentioned above, with great potential for inhibition in COX-2, still do not have in vitro or in vivo studies that evaluate this activity. Therefore, these are important findings for future research and development studies of COX-2 selective anti-inflammatory drugs.

### 2.7. Prediction of Toxicological Properties

LMQC72, LMQC36, and LMQC5 were also submitted for evaluation of their toxicological properties using DEREK software. This assessment was carried out to investigate whether these compounds had a profile of adverse toxicological effects on humans, mice, and rats. According to the results (Table 8), the selected compounds did not present any toxicity alert. Results of the pivot compound Rofecoxib, on the other hand, were flagged as “plausible”, since it presented a warning of hepatotoxicity (humans, mice and rats) for derivatives of the furanone group [63].

Table 8 shows also the oral lethal dose prediction (LD_50_) based on mg/kg body weight and toxicity class ranging from I to VI, performed on the ProTox-II web server (http://tox.charite.de/protox_II). LD_50_ of the LMQC72 structure was 674 mg/kg and of the LMQC50 1400 mg/kg both with IV classification was considered harmful if ingested (300 < LD_50_ ≤ 2000), however, they showed higher lethal dose when compared to rofecoxib. LMQC36 presented a LD_50_ value of 6500 mg/kg and classification VI, which is non-toxic if ingested, estimated as the best result of an oral lethal dose. Therefore, the results for LD_50_ of the investigated compounds are better than the commercial compound and may present greater safety in use [21].

### 2.8. Predictions of the Cardiotoxicity

The compounds were also submitted to the preADMET [18,19] software to assess the cardiotoxicity. Drug candidates often cause an unwanted blockage of the potassium ion channel of the human ether-a-go-go-related gene (hERG). The blockage leads to long QT syndrome (LQTS), which is a severe life-threatening cardiac side effect [89]. The evaluation of this parameter was by means of hERG ([17] takes into account the electro-affinity calculation (EA) of the compounds. The results of the evaluation of the cardiotoxicity capacity for LMQC72, LMQC36, LMQC50, and rofecoxib can be seen in Table 9.

LMQC72, LMQC36, LMQC50, and rofecoxib showed a medium risk of cardiotoxicity in the electro-affinity calculation. This pharmacokinetic property that is related to drug-receptor interaction and electron transfers, we consider an aspect of paramount importance for therapeutic activity and in determining toxicity [90]. The human ether-a-go-go-related gene (hERG) is codified for a protein that forms a voltage-dependent potassium ion channel found in heart and nervous system [91,92,93]; a myocardial conduction disorder (electrical conduction) can alter ventricular repolarization and, consequently, increase the vulnerability for the development of a cardiac action [91]. Therefore, LMQC72, LMQC36, and LMQC50 have a lower risk of cardiotoxicity when compared to rofecoxib, since they do not present violation in the electro-affinity parameter. 

## 3. Materials and Methods 

### 3.1. Template Compound

Crystallographic structure (PDB 5KIR at 2.7 Å resolution) of human cyclooxygenase-2 (hCOX-2) was obtained as PDB file from the Protein Data Bank (PDB) (https://www.rcsb.org/pdb) complexed with the pivot rofecoxib [12,14].

### 3.2. Generation of Confomer Library in Databases

In this step, we used six commercial databases for virtual screening based of rofecoxib ligand: Chembridge DIVERSet™-EXPRESS-Pick™ Collection (DIVERSet™-EXP), DIVERSet CORE Library (DIVERSet™-CL), ZINC drug database, ZINC natural stock e ZINC Drug@FDA BindingDB, and Maybridge. For each molecule in the database, we obtained 300 conformers using the MMFF94 Molecular Force Fields were generated [94], running on OMEGA v3.3.1.2 software (Open Eye Scientific Software, Santa Fe, NM, http://www.eyesopen.com) for Windows 7 operating system and Intel Core i7 machine of 2.4 GHz. Initially, for each molecule in the database, the fast conformer generation method was used with a maximum energy tolerance of 9 kcal.mol^−1^ and mean square deviation (RMSD) of 0.6 Å [15,16,25].

### 3.3. Virtual Screening

#### 3.3.1. Rapid Overlay of Chemical Structures (ROCS)

In this study, Rapid Overlay of Chemical Structures (ROCS) v3.3.2.2 (OpenEye) software was used as a tool for three-dimensional (3D) molecular similarity research. We used six databases to select chemical compounds through the ROCS software (https://www.eyesopen.com/rocs) [30], with Gaussian function algorithm located in atoms that proposes the best overlap between molecules in a characteristic set that can be a steric volume or the molecular interaction, called ComboScore. This was done to generate and score three-dimensional (3D) overlays of the database with the pivot compound (rofecoxib) in order to seek better compounds for the COX-2 receptor, to get the highest rated structures (Top_200) of each base, totaling 12,000 compounds [15,16,25,34] This software generates input files for the EON program.

#### 3.3.2. Electrostatic Similarity (EON)

EON v2.3.2.2 (OpenEye) software is an electrostatics comparison program (https://www.eyesopen.com/eon) [95]—it compared the electrostatic potential maps of pre-aligned molecules and determined the Tanimoto measures for the comparison of the six databases. Moreover, it calculated the new partial load to minimize energy using the MMFF94 force field [94]. Electrostatic classification was based on Tanimoto’s electrostatic scores; the electrostatic arrangement was obtained from the overlapping of positive and negative charges when completing the variation of an identical to negative values. In this study, a lower energy of rofecoxib conformer was used to perform electrostatic comparisons (more rigid conformation, based on the available crystallographic structure). The output files were grouped according to the scores and the results were classified based on “ET combo” analogous to “Tanimoto Combo”. In the end, only the “100 best compounds/base” were selected, affording 600 molecules [15,16,25,34,35,96,97].

### 3.4. In Silico Pharmacokinetic and Toxicological Properties

#### 3.4.1. Pharmacokinetic Predictions

The assessment of a number of key physicochemical properties, pharmacokinetic parameters, and toxicity endpoints was carried out for the compounds that passed the virtual screening step—the Top 100 of each database. Pharmacokinetic (#star, “Rule of Five”, Human intestinal absorption, QPPCaco, QPPMDCK, QPlogP*o*/*w*, CNS, and QPlogBB) properties were predicted using the Schrodinger’s Suite QikProp v.3.5, and Derek Nexus Software 2.0 [25,40]. 

#### 3.4.2. Toxicological Predictions

The toxicity of the compounds with the best pharmacokinetic profiles was assessed using (DEREK) 10.0.2 Nexus program [25,40]. Deductive Estimation of Risk from Existing Knowledge (DEREK) predicts potential toxicity and toxicophoric groups and also includes the following toxicological parameters: carcinogenicity, mutagenicity, genotoxicity, skin sensitization, teratogenicity, irritation, respiratory sensitization, reproductive toxicity [37,63]. 

This software analyses qualitative predictions and, in this way, generates alerts about the possible toxic action of the chemical compounds analyzed. In this step, the compounds were evaluated in aspects involving the types of toxicity and possible toxicophoric effects [37,98]. We have considered DEREK toxicity alerts involving the human species and also classified as plausible in mammals, but compounds containing any toxicophoric groups were also discarded [20], through visual inspection using the Maestro 9.9 program.

#### 3.4.3. Prediction of Toxicity Lethal Dose (LD_50_)

The selected compounds were submitted to the ProTox web server (http://tox.charite.de/protox_II) [99], which identifies lethal oral doses (LD_50_) [88]. The prediction method is based on the analysis of the two-dimensional (2D) similarity to compounds with known LD_50_ values and the identification of fragments over-represented in toxic compounds. The results are generated instantly on the server page, showing the predicted average lethal dose (LD_50_) in mg/kg of weight and the toxicity classes (I, II, III, IV, V and VI) [21,98].

#### 3.4.4. Prediction of the Cardiotoxicity

The prediction of cardiotoxicity was determined using the online server PreADMET (https://preadmet.bmdrc.kr/) [18] and QikProp [100] software. The prediction method for the risk of cardiac toxicity is based on the inhibition property of the human ether-to-go-go (hERG) gene based on the electron affinity of the compounds. PreADMET instantly generates alerts on the server page classified as: low risk, medium risk, and high risk for the hERG property [17]. 

### 3.5. Prediction of Biological Target

This screening step for the prediction of the biological target was performed via web servers: Molinspiration and SwissTargetPrediction. The bioactivity score of selected compounds was evaluated using the Molinspiration Server Cheminformatics tool (http://www.molinspiration.com) [101]. The prediction made was based on the enzyme inhibition score, taking into account the pivot molecule. The results are analyzed according to Roy; Samant; Chowdhary [102]. Therefore, it is recommended that if the value is equal to or greater than 0.00, the more active it will be, while if the values are between −0.50 and 0.00, it is moderately active, and, if the score is less than −0.50, it will be considered inactive [21,103]. 

Then, the query structures were submitted to the SwissTargetPrediction web server (http://www.swisstargetprediction.ch), to predict small molecule protein targets in *Homo sapiens* (Top_25). Targets are classified according to their percentage probability on the assumption that if the molecule is active, it is likely to bind to some protein. The investigation of the bioactivity target was based on the value of the enzymatic target of the pivot molecule rofecoxib with known bioactivity. The results of the server prediction via SwissTargetPrediction web server are presented as a percentage in a pie chart [22,64].

### 3.6. Molecular Docking Simulations Study

#### 3.6.1. Selection of Therapeutic Target Structure and Ligand

Molecular docking simulations were based on fitting the ligand to the active site of an enzyme. This simulation is called re-docking which aims to recover, from computer simulation, the original position of a ligand present in a crystallographic structure of a protein-ligand complex [59]. For the determination of this protocol an approach called validation is used, where we used as reference a crystallographic structure already determined [62,83].

For this study, the crystallographic structure of *h*COX-2 complexed with rofecoxib ligand deposited in the PDB was used with code 5KIR (*Homo sapiens*) and a resolution of 2.7 Å [11,50]. The enzyme structure was prepared by removing water and binders, and adding hydrogen atoms, using Discovery Studio 4.1 software. Then, the AutoDock/Vina software was subjected to molecular coupling [62,104,105].

#### 3.6.2. Docking Study with AutoDock 4.2/Vina 1.1.2 Via Graphical Interface PyRx (Version 0.8.30)

Molecular docking calculations were performed using AutoDock 4.2/Software Vina 1.1.2 and the PyRy interface version 0.8. AutoDock is a set of tools that allow the interaction between ligand and macromolecule and provides combinations with algorithm options: Simulated Annealing (SA), Genetic Algorithm (GA), and Lamarckian Genetic Algorithm (LGA). In this work, the search algorithm used was LGA (Lamarckian Genetic Algorithm), that presents the best results in the search for the global minimum [62,83].

The interactions between inhibitors and the receptor were visualized using the Discovery Studio 4.1 software with standard parameters. The evaluation of the molecular coupling was determined by means of the ligand obtained experimentally and the theoretical conformation performed with the molecular coupling in the PDB (5KIR), and were validated by the RMSD value. Table 10 shows the x, y and z coordinates according to the interaction between COX-2 and the standard ligand. The x, y and z coordinates of the receivers were determined according to the average region of the active site. Moreover, ten solutions were calculated for each ligand and minimum conformations of binding energy were analyzed [15,16,20,25,59].

The energy scoring function was used to assess the free binding energy (ΔG) of interactions between COX and ligands in PyRx 0.8.30. The analysis of the poses (conformation + orientation) of the binders was also taken into account in the selection of the best binding free energy and binding affinity calculations in AutoDock 4.2/Vina 1.1.2 in order to assess selectivity towards *Homo sapiens* as a function of binding affinity at the COX-2 receptor.

### 3.7. Molecular Dynamics (MD) Simulation Protocol

The initial structure for the system was obtained from molecular docking methods, as described in the previous section. The restrained electrostatic potential (RESP) protocol with the HF/6-31G* basis sets was applied to obtain the charges of the atoms of each ligand [106,107,108] Atomic charge calculated using Gaussian [81,109,110] The parameters of the ligand were constructed with the Antechamber module, available in the Amber16 package [111,112,113]. The protonation state of ionizable residues of protein structure was analyzed using the PROPKA [114] server in the neutral pH before performing the MD simulations. The ligand was treated with the General Amber Force Field (GAFF) and protein was treated with the ff14SB [10]. The force field parameters developed by Giammona were used for the heme group [115]. The system was constructed for the simulation using the tutorial for the LEaP (tLEaP) of Amber 16 package. The system was solvated in an octahedron periodic box containing water molecules in the TIP3P model [116]. The partial charges of the systems were neutralized by adding counterions. 

We used the sander.MPI for the four stages of energy minimization. In each of these stages, it took 3000 cycles using the steepest descent method and 5000 cycles using the conjugate gradient algorithm. In the first stage the hydrogen atoms of the water molecules were optimized; then, the ions and the water molecules were minimized; in the third stage, the hydrogen atoms of the protein, and in the last step, the solute and the solvent, underwent the process of energy minimization.

Three heating steps were used for a total time of 800 picoseconds to raise the system temperature to 300 K. First, the solute was restricted with a constant harmonic force of 25 kcal mol^−1^. Å^−2^, so only the solvent and the counter ions moved. In the next step, the constant harmonic force was removed. To equilibrate the systems, we performed 2 ns simulations with no restriction at constant temperature. Finally, for each system, we performed 150 ns of molecular dynamics of production. Particle Mesh Ewald method [117] was used for the calculation of electrostatic interactions and the bonds involving hydrogen atoms were restricted with the SHAKE algorithm [118]. Temperature control was performed with the Langevin thermostat [119] within collision frequency of 2 ps-1.

### 3.8. Free Energy Calculation Using MM/GBSA Approach

To estimate the binding affinity (ΔG_bind_), we used the Molecular Mechanics/*G*eneralized Born Surface Area (MM-GBSA) method [80,120,121]. The ΔG_bind_ was calculated according to the following equations:

ΔG_bind_ = ΔG_complex_ − ΔG_receptor_ − ΔG_ligand_ (1)

ΔG_bind_ = ΔH − TΔS ≈ ΔE_MM_ + ΔG_solv_ − TΔS (2)

ΔE_MM_ = ΔE_internal_ + ΔE_ele_ + ΔE_vdW_ (3)

ΔG_solv_ = ΔG_GB_ + ΔG_NP_ (4)

The affinity energy (ΔGbind) is the summation of the interaction energy of the gas phase between protein-ligand (ΔEMM), desolvation free energy (ΔGsolv) and system entropy (-TΔS). ΔEMM is the result of the sum of internal energy (ΔEinternal, sum of the energies of connection, angles, and dihedral) electrostatic contributions (ΔEele) and the van der Waals term (ΔEvdW). ΔGsolv is the sum of the polar (ΔGGB) and non-polar (ΔGNP) contributions. ΔGSA was determined from the solvent accessible surface area (SASA) estimated by the linear combination of pairwise overlaps (LCPO) algorithm.

### 3.9. Per-Residue Energy Decomposition

The MM/GBSA method was used to determine the energy contribution of each protein residue, thus, rendering it possible to determine which residues are most important for the ligand interaction with the active site. The interaction energy of residues with the inhibitor can be described from four terms: van der Waals contribution (ΔE_vdW_), electrostatic contribution (ΔE_ele_), polar solvation contribution (ΔG_pol_), and nonpolar solvation contribution (ΔG_nonpol_), according to the equation [25,122,123]:

ΔG_ligand-residue_ = ΔE_vdW_ + ΔE_ele_ + ΔG_pol_ + ΔG_nonpol_ (5)

## 4. Conclusions

In this study, a computational strategy was applied to identify new potential selective hCOX-2 inhibitors base Conclusions d on the known drug rofecoxib. Compounds from six databases were filtered by a ligand-based virtual screening study, followed by pharmacokinetic, toxicological, and molecular dynamic studies. The selected structures LMQC72, LMQC36, and LMQC50 have aspects strictly related to physical-chemical properties and biological activity. Therefore, such selected structures reproduce values within the limits established in the pharmacokinetic predictions: absorption and distribution in the human body. Moreover, in the prediction of toxicity the structures LMQC72, LMQC36, and LMQC50 did not present alerts for possible toxic groups.

Through the study of molecular dynamics, LMQC72, LMQC36, and LMQC50 were identified as promising due to values of affinity energy relatively close to those obtained for rofecoxib. Along the trajectories of molecular dynamics simulations, the selected compounds showed conformational stability, as well as the pivot compound. LMQC72, LMQC50, and LMQC36 showed satisfactory pharmacokinetic results related to absorption and distribution. The toxicological predictions of these compounds did not display alerts for possible toxic groups and lower risk of cardiotoxicity compared to rofecoxib. This demonstrates that LMQC72, LMQC36, and LMQC50 can be considered as putative *h*COX-2 inhibitors, in addition to serving as the basis for the new anti-inflammatory drug project.

## Figures and Tables

**Table 1:** Pharmacokinetic properties of the selected compounds.

Structures	#Stars ^a^	EA (eV) ^b^	RO5 ^c^	%HOA ^d^	QplogP*o/w* ^e^	QPPCaco ^f^	QPP MDCK ^g^	CNS ^h^	Qplog BB ^i^
Normal range	0.0 to 5.0	−0.9 to 1.7	Max. 4	0 to 100	−2.0 to 6.5	<25 poor >500 great	<25 poor >500 great	−2 (inactive) +2 (active)	−3.0 to −1.2
Rofecoxib	1	1.99	0	82.40	1.45	420.96	194.20	−1	−0.81
LMQC72	0	1.37	0	100.00	2.18	1470.77	900.66	0	−0.17
LMQC36	0	0.73	0	100.00	3.75	1751.71	2254.41	0	−0.07
LMQC50	0	1.44	0	100.00	4.21	13,737.5	3415.52	0	−0.77

^a^ Number of computed properties which fall outside the required range for 95% of known drug; ^b^ electronic affinity (EA); ^c^ number of violations of Lipinski’s ‘Rule of Five’ (RO5); ^d^ percentage of human oral absorption (%HOA); ^e^ apparent permeability of compound between octanol/water (QplogP*o*/*w*); ^f^ permeability of the differentiated cells of intestinal epithelium Caco-2 (QPPCaco); ^g^ Madin–Darby canine kidney (QPPMDCK); ^h^ activity in the central nervous system; ^i^ apparent permeability of compound in the blood-brain barrier [38].

**Table 2:** Comparison between experimental and theoretical binding affinities.

Enzyme	Ligand	Experimental Binding Affinity (kcal/mol) ^a^	Ki (nM)	Docking Predicted Binding Affinity (kcal/mol)	Resolution
*h*COX-2 (PDB 5KIR)	Rofecoxib (RCX)	−9.2 [14]	310	−10.4	2.69 Å [14]

^a^ The values calculated from the experimentally determined inhibition constant (Ki), found in the Protein Data Bank (PDB), according to the Equation: ΔG = R.T.lnKi, were R (gas constant) = 1.987.10^−3^ kcal/(mol^−1^.K^−1^) and T (temperature) = 310 K [61].

**Table 3:** Type of interactions and interacting residues of *h*COX-2 and rofecoxib.

Molecular Docking	Residues	Distance (Å)	Type	∆G (kcal/mol)
Rofecoxib vs. 5KIR	His90	2.64213	Hydrogen Bond	−10.4
	Val349	4.41806	Pi-Alkyl	
	Leu352	5.44011	Pi-Alkyl	
	Arg513	2.55259	Carbon Hydrogen Bond	
	Arg513	3.07173	Carbon Hydrogen Bond	
	Arg513	2.37819	Hydrogen Bond	
	Phe518	5.82249	Pi-Pi Stacked	
	Val523	3.80966	Pi-Alkyl	
	Ala527	4.95368	Pi-Alkyl	
	Ala527	3.97589	Pi-Alkyl	
	Ala527	2.61541	Carbon Hydrogen Bond	
	Ser530	2.84906	Carbon Hydrogen Bond	

**Table 4:** Type of interactions and interacting residues of *h*COX-2 and compounds LMQC72, LMQC36, LMQC50.

Molecular Docking	Residues	Distance (Å)	Type	∆G (kcal/mol)
	Leu352	2.182419	Hydrogen Bond	
	Ser353	2.904348	Hydrogen Bond	
LMQC72 vs. 5KIR	Phe518	2.256368	Hydrogen Bond	
	Gln192	2.826605	Hydrogen Bond	−11.0
	Val523	3.868765	Pi-Alkyl	
	Met522	4.589701	Alkyl	
	Ala527	4.200118	Pi-Alkyl	
	Ala527	3.367419	Pi-Sigma	
	Val349	3.784354	Pi-Sigma	
	Val349	4.593121	Alkyl	
	Leu351	4.422239	Alkyl	
LMQC36 vs. 5KIR	Leu352	3.576441	Carbon Hydrogen Bond	
	Val523	3.520289	Pi-Sigma	−10.6
	Val523	5.175953	Pi-Alkyl	
	Ser353	2.921642	Carbon Hydrogen Bond	
	Arg513	4.805970	Pi-Cation	
	Gly192	3.057449	Carbon Hydrogen Bond	
	Ile527	4.774353	Alkyl	
	Phe518	4.346549	Pi-Alkyl	
	Leu351	5.018402	Alkyl	
	Leu359	4.885905	Alkyl	
	Val116	5.139531	Alkyl	
	Try355	2.926941	Hydrogen Bond	
	Val349	5.180672	Pi-Alkyl	
LMQC50 vs. 5KIR	Ser353	3.396209	Pi-Sigma	
	Val523	3.868112	Pi-Sigma	
	Leu352	2.176448	Hydrogen Bond	−10.2
	Arg513	3.513371	Carbon Hydrogen Bond	
	Phe518	5.858526	Pi-Pi Stacked	
	Gly526	4.175181	Amide-Pi Stacked	
	Met522	4.690481	Alkyl	
	Ala527	3.972980	Pi-Sigma	
	Arg120	2.589874	Hydrogen Bond	

**Table 5:** Selected structures resulting from the molecular docking.

Number	Compounds	Code ID and Database	Chemical Identification	Code SMILES
LMQC72	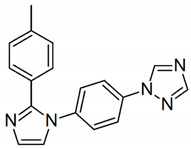	Chembridge_DIVERSet-CL ZINC 72149848	C_18_H_15_N_5_ 1-{4-[2-(4-methylphenyl)-1H-imidazol-1-yl]phenyl}-1H-1,2,4-triazole	Cc1ccc(cc1)c4nccn4c2ccc(cc2)n3cncn3
LMQC36	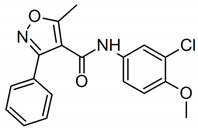	Chembridge_DIVERSet-EXP ZINC3615660	C_18_H_15_N_5_ *N*-(3-chloro-4-methoxyphenyl)-5-methyl-3-phenyl-1,2-oxazole-4-carboxamide	COc1ccc(cc1Cl)NC(=O)c3c(C)onc3c2ccccc2
LMQC50	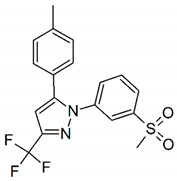	Drug@FDA_BindingDB Binding_DB 50224	C_18_H_15_N_5_ *N*-(3-chloro-4-methoxyphenyl)-5-methyl-3-phenyl-1,2-oxazole-4-carboxamide 4-(5-(p-tolyl)-3-(trifluoromethyl)-1H-pyrazol-1-yl) benzenesulfonamide	CS(=O)(=O)c1cccc(c1)n3nc(cc3c2ccc(C)cc2)C(F)(F)F

**Table 6:** Prediction of bioactivity of the selected compounds.

Compound	GPCR Ligand	Ion Channel Modulator	Kinase Inhibitor	Nuclear Receptor Ligand	Protease Inhibitor	Enzyme Inhibitor	Enzyme (%) ^a^
Rofecoxib	0.20	0.13	0.16	−0.37	−0.14	0.61	32%
LMQC72	0.19	−0.41	−0.23	−0.02	−0.45	0.07	16%
LMQC36	−0.29	−0.40	0.04	−0.04	−0.10	−0.43	8%
LMQC50	0.03	−0.18	−0.18	0.12	0.26	0.21	4%

^a^ Web Swiss Target Prediction: probability (%) for the query molecule—assumed as bioactive—to have this enzyme as target [64].

**Table 7:** Binding free energy *h*COX-2-ligands.

Compound	ΔEvdW ^a^	ΔEele ^b^	ΔGGB ^c^	ΔGNP ^d^	ΔGbind ^e^
Rofecoxib	−48.12	−23.66	35.74	−9.27	−45.31
LMQC72	−52.92	−21.45	42.71	−6.92	−38.58
LMQC36	−45.93	−7.79	23.28	−5.66	−36.10
LMQC50	−49.80	−13.86	29.93	−5.67	−39.40

^a^ Variation of Van der Waals energy; ^b^ Variation of electrostatic energy; ^c^ Variation of polar energy; ^d^ Variation of non-polar energy; ^e^ binding Energy.

**Table 8:** Predictions of the toxicological properties the selected compounds.

Compounds	Prediction Alert	Toxicophoric Group	Toxicity Alert	LD_50_	Toxicity Class ^a^
Rofecoxib	Hepatotoxicity in human, mouse and rat	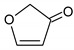	Plausible	4500 mg/kg	V
LMQC72	-	-	No alerts	674 mg/kg	IV
LMQC36	-	-	No alerts	6500 mg/kg	VI
LMQC50	-	-	No alerts	1400 mg/kg	IV

^a^ Class I: lethal if swallowed (LD_50_ ≤ 5); Class II: lethal if swallowed (5 < LD_50_ ≤ 50); Class III: toxic if swallowed (50 < LD_50_ ≤ 300); Class IV: harmful if swallowed (300 < LD_50_ ≤ 2000); Class V: may be harmful if swallowed (2000 < LD_50_ ≤ 5000) e Class VI: non-toxic (LD_50_ > 5000) [88].

**Table 9:** Predictions of the cardiotoxicity properties of the selected compounds.

Compound	QpLog hERG ^a^	EA (eV) ^b^
Rofecoxib	Medium risk	1.997
LMQC72	Medium risk	1.374
LMQC36	Medium risk	0.739
LMQC50	Medium risk	1.446

^a^ PreADMET software: QpLog: low, medium and high [19]; ^b^ QikProp software: Electron affinity: −0.9 to 1.7 [38].

**Table 10:** Protocol data used in the validation of molecular docking.

Enzyme	Ligand	Coordinates of the Grid Center	Grid Size (Points)
COX-2 (PDB code: 5KIR) *Homo sapiens*	Rofecoxib	X = 24.065 Y = 40.416 Z = 3.057	17 x 20 y 27 z
